# The effects of substituting organic fertilizer for nitrogen fertilizer on seed cotton yield and soil enzyme activity under drip irrigation

**DOI:** 10.3389/fpls.2026.1804515

**Published:** 2026-04-02

**Authors:** Rui Zhou, Pingchuan Ma, Long Ma, Shenggao Song, Shuangqing Lv, Chunming Chi, Lili Yang

**Affiliations:** 1 College of Agriculture, Tarim University, Alar, China; 2 Key Laboratory of Genetic Improvement and Efficient Production for Specialty Crops in Arid Southern Xinjiang of Xinjiang Corps, Alar, China; 3 Key Laboratory of Saline-alkali Soil Improvement and Utilization (Saline-alkali land in arid and semi-arid regions), Ministry of Agriculture and Rural Affairs, Urumqi, China

**Keywords:** cotton yield, fiber quality, nitrogen accumulation, organic fertilizer, soil enzyme activity

## Abstract

**Introduction:**

The application of organic fertilizers can increase carbon and nitrogen sources in the soil. This could affect the quantity of microorganisms and the metabolism of nutrients in the soil, thereby influencing soil enzyme activity and cotton yield. This study explored the effects of substituting organic fertilizer for different proportions of nitrogen fertilizer on cotton yield, fiber quality, and soil enzyme activity.

**Methods:**

The experiment involved a field trial in Xinjiang with a total of 11 treatments. T1 was the no-fertilizer treatment (control), T2 was the single fertilizer treatment (chemical fertilizer only), and T3–T11 were the combined fertilizer treatments (three different types of organic fertilizers replacing 10%, 20%, and 30% of nitrogen fertilizer).

**Results:**

T11 achieved the highest plant height and fruit shoot number, while T8 and T10 respectively contributed to the greatest leaf SPAD and ginning outturn. The combined fertilizer treatments significantly increased cotton yield and fruit shoot number compared with the control and single fertilizer treatment. When different types of organic fertilizers were applied, there were no significant differences in seed cotton yield among T5, T8, and T11 (30% nitrogen replacement). The yield reached 6243.05 (T5), 6661.11 (T8), and 6933.47 (T11) kg ha^-1^, being 59%, 70%, and 77% higher than that of the single fertilizer treatment (T2). Fiber length, uniformity, and strength were all highest in T11. Both cotton biomass and nitrogen accumulation were enhanced under the combined fertilizer treatments. In particular, T5, T8, and T11 increased soil microbial biomass carbon and nitrogen contents. T5 stimulated soil β-xylotetraerase activity; T8 elevated β-1,4-N-acetylamino glucosaminidase, cellulose β-glucosidase, and β-1,4-glucosidase activities; T11 improved alkaline phosphatase activity. The comprehensive enzyme activity index of T8 was significantly higher than other treatments.

**Conclusion:**

Substituting liquid organic fertilizer for 30% of nitrogen fertilizer (T8 and T11) is the most suitable strategy for cotton planting in Xinjiang.

## 1 Introduction

Cotton (*Gossypium hirsutum* L.) is the main source of natural fibers, providing 35% of the fibers worldwide (Abdelraheem et al., 2019). Therefore, cotton planting plays a crucial role in the global clothing industry and textile sector. China contributes one-fourth to the world’s total cotton production (Yang et al., 2021). Xinjiang is located in the arid and semi-arid region of China, serving as a major cotton-growing area (Feng et al., 2017). The planting area and output of cotton in Xinjiang account for 67% and 85% of the China total, respectively (Bai et al., 2021). However, the natural endowment for cotton growth is insufficient in many areas of Xinjiang, where the cultivated soil generally suffers from salinization, drought, and nutrient deficiency (Wang et al., 2018; Lu et al., 2022). These adverse factors severely restrict the improvements in cotton yield and fiber quality. Moreover, some cotton farmers lack scientific concepts and techniques for soil nutrient management. The blindness in their fertilization decisions further aggravates the negative impact on cotton yield and fiber quality (Li et al., 2020).

The yield and fiber quality of cotton are co-regulated by multiple factors, such as genetic characteristics, environmental conditions, and water and fertilizer management (Zhu et al., 2023). Rational water and fertilizer management is essential for cotton planting in arid regions with low soil fertility and scarce water resources (Zaman et al., 2021). Numerous studies have demonstrated the pronounced influence of nitrogen management on cotton yield and fiber quality (Luo et al., 2018; Liu et al., 2021a). Nitrogen deficiency causes premature senescence in crops, while nitrogen overuse leads to excessive plant growth and late maturity (Dong et al., 2012). Insufficient nitrogen supply at the flowering stage of cotton may result in substantial yield losses. An appropriate amount of nitrogen fertilizer can enhance cotton yield by increasing the number and weight of cotton bolls (Shah et al., 2017; Luo et al., 2020). Nitrogen fertilizer also has a strong impact on cotton fiber quality, whereby nitrogen deficiency reduces fiber length and strength (Read et al., 2006). Rochester et al. (2001) reported that increasing nitrogen fertilizer application improved fiber length and strength in cotton. However, Ma et al. (2022) found that with increasing nitrogen content, cotton fiber length and strength first increased and then decreased. These findings underscore the importance of applying a suitable amount of nitrogen fertilizer to improve seed cotton yield and fiber quality.

Many farmers fail to accurately determine the appropriate amount of chemical nitrogen fertilizer for cotton production, with excessive use in many cases (Feng et al., 2023; Hou et al., 2022). This not only compromises crop growth but also leads to nutrient waste, potentially causing environmental pollution (Khan et al., 2017; Tsakmakis et al., 2019; Hedayati et al., 2019). In 2015, China launched the Zero Growth Action of Chemical Fertilizers. This action advocates the use of organic fertilizers to replace chemical fertilizers and the adoption of integrated water and fertilizer management techniques to enhance fertilizer utilization while reducing chemical fertilizer application. In addition to providing rich nutrient supply for crops, organic fertilizers also enhance soil microbial activity and optimize soil physical-chemical properties, creating a more suitable environment for crop growth. However, the use of organic fertilizers has some limitations, such as the need for microbial mineralization and decomposition before plant absorption and utilization. Additionally, solid organic fertilizers are difficult to adapt to the application of integrated water and fertilizer management. Partially substituting chemical fertilizers with organic fertilizers shows complementary advantages, and their co-application can maximize the benefits of single fertilizers.

The substitution of chemical fertilizers with organic fertilizers is regarded as an effective strategy to reduce chemical fertilizer use, increase crop yields, and improve crop quality. Wang et al. (2023) showed that replacing 20% of chemical nitrogen fertilizer with organic fertilizer increased crop yield, alongside improved soil nitrogen availability, elevated bacterial and fungal abundances, and enhanced enzyme activity. Sun et al. (2025) found that replacing 20% of chemical fertilizer with organic fertilizer enhanced protein content in potato tubers mainly by increasing soil urease activity and leaf chlorophyll content. However, the effects of different types of organic fertilizers may vary due to distinct nutrient absorption patterns of crops and heterogenous physical-chemical properties of soils. Despite extensive research on the substitution of chemical fertilizers with organic fertilizers in China, there is still a paucity of relevant studies for cotton under drip irrigation in Xinjiang. Furthermore, liquid organic fertilizers show great development potential and can meet the needs of sustainable modern agriculture. Currently, the actual effects of liquid organic fertilizers in replacing chemical fertilizers remain poorly understood.

Previous studies have shown that using organic fertilizer in place of 20% of nitrogen fertilizer is the optimal substitution ratio (Wang et al., 2023; Sun et al., 2025). We hypothesized that substituting organic fertilizer for 20% of chemical nitrogen fertilizer is the suitable strategy for cotton production in drip-irrigated fields in Xinjiang. To test the hypothesis, this study designed a field trial involving three different types of organic fertilizers and three substitution ratios (10%, 20%, 30%) for chemical fertilizers. The aim of this study was to identify the suitable type of organic fertilizers and substitution ratio of chemical fertilizer for cotton fields under drip irrigation in Xinjiang. This research could provide empirical evidence and practical guidance for high-quality and high-yield cotton cultivation in arid regions.

## 2 Materials and methods

### 2.1 Experimental site

In 2024, the experiment was conducted in Alar (40°35′21.80" N, 81°19′40.37" E), Xinjiang Uygur Autonomous Region, China. The experimental site has a temperate continental climate with an average annual temperature of 12.4°C. The average annual precipitation ranges from 40.1 to 82.5 mm, and the annual sunshine duration is over 2900 hours. Before the start of the experiment, the 0–20 cm soil had a pH value of 8.29 and contained 6.26 g kg^-1^ of organic matter. The contents of available nitrogen, phosphorus, and potassium were 38.48 mg kg^-1^, 19.60 mg kg^-1^, and 164 mg kg^-1^, respectively.

### 2.2 Experimental design

This experiment used a randomized block design with 11 treatments: T1—blank control treatment, without adding any fertilizers; T2—single fertilizer treatment with 300 kg N ha^-1^; T3, T4, and T5—solid organic fertilizer replacing 10%, 20%, and 30% chemical nitrogen fertilizer, respectively; T6, T7, and T8—liquid organic fertilizer 1 replacing 10%, 20%, and 30% chemical nitrogen fertilizer, respectively; T9, T10, and T11—liquid organic fertilizer 2 replacing 10%, 20%, and 30% chemical nitrogen fertilizer, respectively. Each treatment was replicated three times, with a total of 33 plots. The plot size was 28 m^2^ each (4 m wide × 7 m long) and an interval of 0.5 m was established between adjacent plots. Urea (46% N) was applied as the chemical nitrogen fertilizer. The total nitrogen content in the solid organic fertilizer was 13.14%, and the liquid organic fertilizers contained 180 g N L^-1^ (1) and 20 g N L^-1^ (2). Triple superphosphate (18% N, 46% P_2_O_5_) and potassium sulfate (50% K_2_O) were applied as phosphate fertilizer (150 kg ha^-1^) and potassium fertilizer (120 kg ha^-1^), respectively. The local cotton variety ‘Xinlu Zhong 46’ was used in the experiment. The nitrogen fertilizer was applied in the ratio of 4:1:1:1:1:1:1, and the phosphorus and potassium fertilizers were applied equally in seven splits. Other field management practices were consistent with the convention of local farmers.

### 2.3 Sample collection and analysis

#### 2.3.1 Plant sampling and analysis

During the cotton ripening stage, 15 uniformly growing plants were randomly harvested from each plot. Plant height, leaf chlorophyll content (SPAD value), and stress resistance related parameters (catalase, CAT; malondialdehyde, MDA) were measured. The number of fruiting shoots, cotton fiber quality, and raw cotton yield were also recorded. Whole plant samples were weighed in five parts: roots, stems, leaves, cotton exocarp, and seed cotton. A portion of the samples was subjected to 90 °C blanching for 30 min and then dried at 65 °C until constant weight to determine the dry matter content. Another portion of the dried samples was ground, digested using H_2_SO_4_-H_2_O_2_, and then analyzed by the Kjeldahl method to quantify the total nitrogen content.

#### 2.3.2 Soil sampling and analysis

During the cotton ripening stage, soil samples (0–20 cm) were collected at five points in each plot. The samples were pooled and a portion was stored at 4 °C for the determination of soil microbial biomass carbon (MBC), microbial biomass nitrogen (MBN), and key enzyme activities. MBC and MBN were determined using the chloroform fumigation-K_2_SO_4_ extraction method. The extracted samples were analyzed using a TOC-L analyzer (CPH, Shimadzu, Japan) and a flow analyzer (AA3, Seal, Germany). The microplate fluorescence method (Deforest, 2009) was employed to assay the activities of β-1,4-glucosidase (BG), cellulose β-glucosidase (CBH), and β-xylotetraerase (BXYL) related to carbon metabolism; β-1,4-N-acetylamino glucosaminidase (NAG) related to nitrogen metabolism; and alkaline phosphatase (AKP) related to phosphorus metabolism. The geometric mean of soil enzyme activities (GME), a comprehensive enzyme activity index, was calculated as follows:


$$GME={\left(X_{1}\times X_{2}\times X_{3}\times \cdots \times X_{n}\right)}^{\frac{1}{n}}$$

where *X_i_* represents the activity of enzyme *i* in the soil (*i* = 1, 2, 3, …, *n*).

### 2.4 Data processing and analysis

Data collation was conducted using Excel 2022 (Microsoft Corp., Redmond, WA, USA). To identify the differences between means, the one-way ANOVA followed by Least Significant Difference (LSD) test was performed in Data Processing System 9.5 (Ruifeng Information Technology, Tianjin, China). A statistically significant difference was deemed to exist at p < 0.05. IBM SPSS Statistics 26 (IBM Inc., Armonk, NY, USA) was used for data variance Pearson analysis. OriginPro 2018 (OriginLab Corp., Northampton, MA, USA) was used for graphical illustration.

## 3 Results

### 3.1 Effect of organic fertilizer replacing nitrogen fertilizer on seed cotton yield

**Figure 1:**
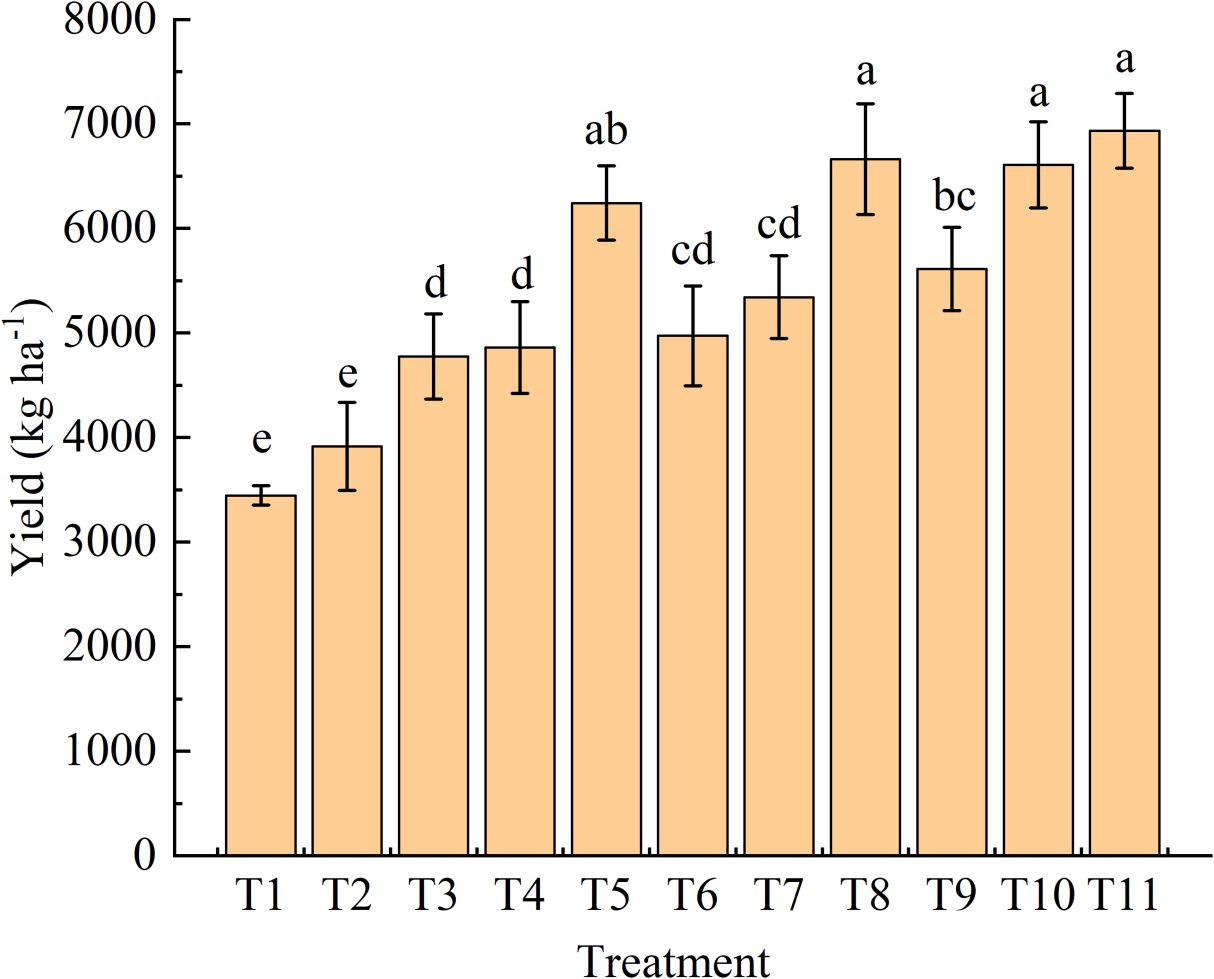
Seed cotton yield under organic fertilizer substitution for nitrogen fertilizer. T1, Blank control treatment with no fertilizer added; T2, Single fertilizer treatment with 300 kg N ha^-1^; T3, T4, and T5, Solid organic fertilizer replacing 10%, 20%, and 30% of chemical nitrogen fertilizer, respectively; T6, T7, and T8, Liquid organic fertilizer 1 replacing 10%, 20%, and 30% of chemical nitrogen fertilizer, respectively; T9, T10, and T11, Liquid organic fertilizer 2 replacing 10%, 20%, and 30% of chemical nitrogen fertilizer, respectively. Error bars indicate standard error of the mean (*n =* 3). Different letters above the error bars indicate significant difference between treatments at p = 0.05 level. The same as below.

Compared with the single fertilizer treatment (T2), all combined fertilizer treatments significantly increased seed cotton yield (**Figure 1**). The highest yield was achieved in T11 (6933.47 kg ha^-1^), which was 77.09% higher than that of T2. There was no significant difference in the yield of T11 compared with T5, T8, and T10. For the same type of organic fertilizers, the cotton yield showed an upward trend with increasing substitution ratio. The liquid organic fertilizers had a slightly greater yield-increasing effect than the solid organic fertilizer at the same substitution ratio.

### 3.2 Responses of cotton plant height and fruiting shoot number to organic fertilizer replacing nitrogen fertilizer

**Figure 2:**
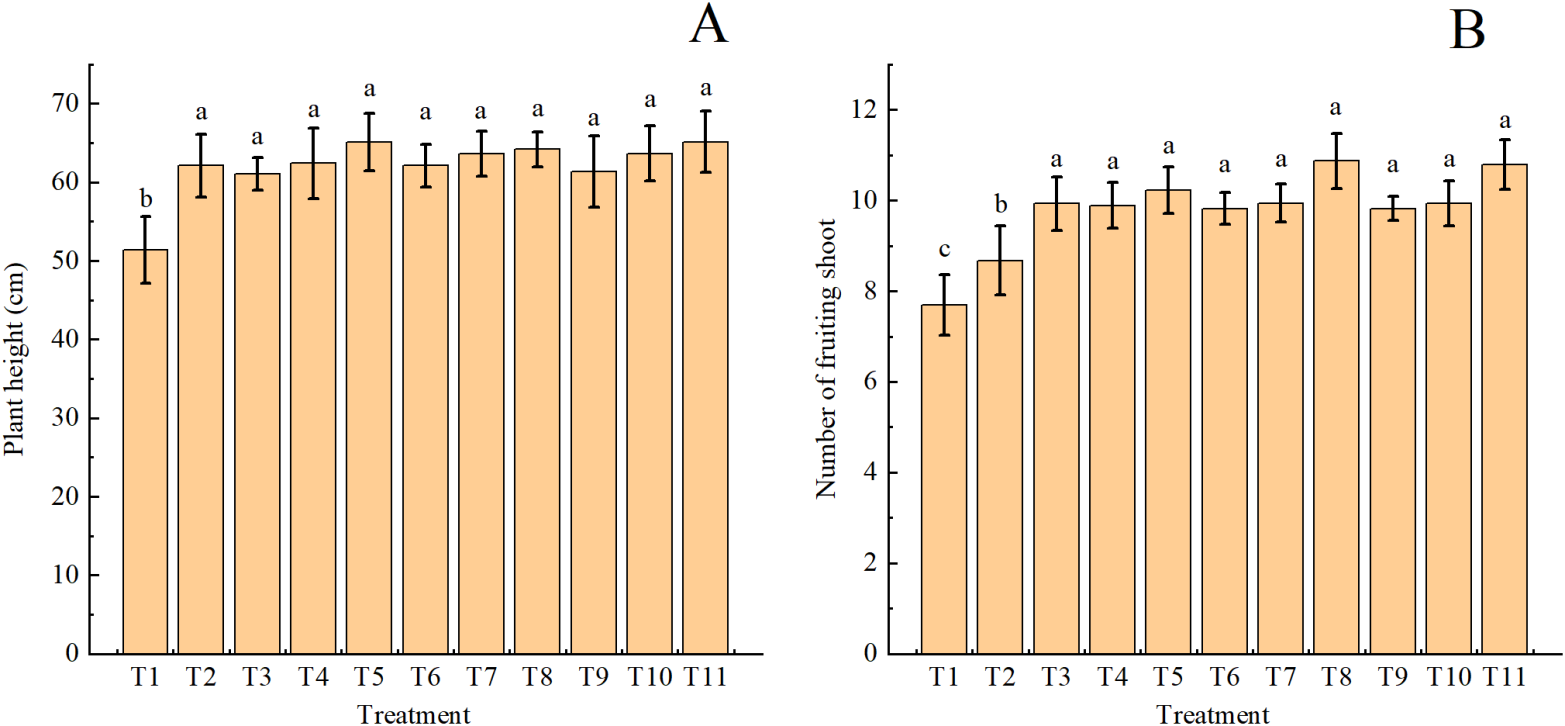
Plant height **(A)** and the number of fruiting shoots **(B)** in drip-irrigated cotton under organic fertilizer substitution for nitrogen fertilizer.

Single and combined fertilizer treatments all significantly improved the height of cotton plants compared with the control (T1; **Figure 2A**). However, there were no significant differences in plant height among various fertilizer treatments. The number of fruiting shoots significantly increased across all combined fertilizer treatments compared with T2 (**Figure 2B**). The effects of combined fertilizer treatments were comparable on the number of fruiting shoots, irrespective of the fertilizer type and substitution ratio.

### 3.3 Variations in leaf chlorophyll content and ginning outturn with organic fertilizer replacing nitrogen fertilizer

**Figure 3:**
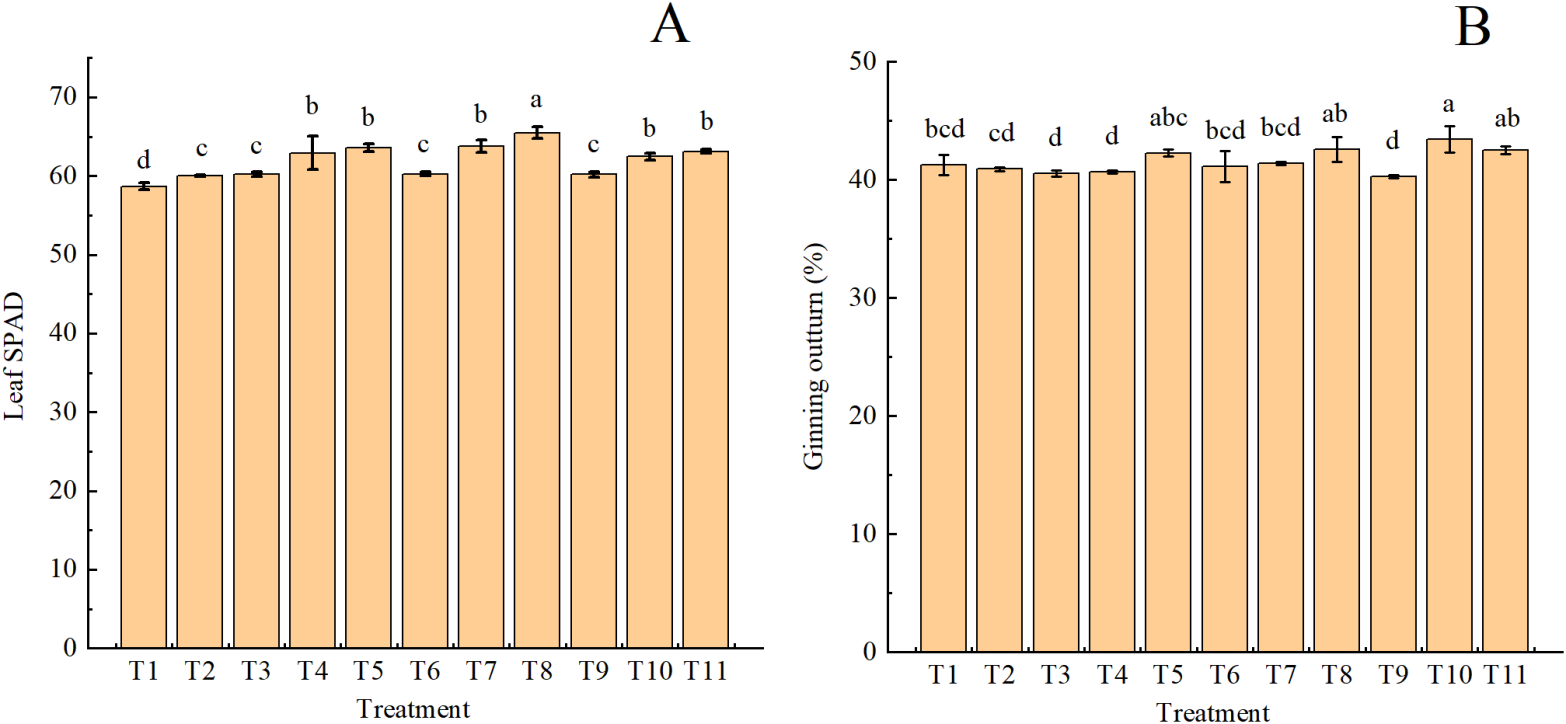
Leaf chlorophyll content (SPAD value; **(A)**) and ginning outturn **(B)** in drip-irrigated cotton under organic fertilizer substitution for nitrogen fertilizer.

The SPAD values of leaves under all fertilizer treatments significantly increased compared with that of T1 (**Figure 3A**). The highest leaf SPAD value was observed in T8, which was significantly higher than that of other treatments. Leaf SPAD values trended upward when the same type of organic fertilizers were used at a greater substitution ratio. T4, T5, T7, T8, T10, and T11 resulted in significantly higher leaf SPAD values than other treatments. T10 showed the highest ginning outturn compared with other treatments (except T5, T8, and T11; **Figure 3B**). In most cases (except T11), ginning outturn exhibited an upward trend with increasing substitution ratio for the same type of organic fertilizers.

### 3.4 Alterations in cotton fiber quality with organic fertilizer replacing nitrogen fertilizer

Among all treatments, T11 showed the highest fiber length, uniformity, and strength, while T10 achieved the greatest micronaire value and fiber elongation rate (**Table 1**). Fiber length was significantly higher in T11 than in T1, T3, T7, and T10. Fiber uniformity had significant difference between T11 versus T1, T6, T8, and T10. The fiber strength in T11 was significantly higher than that of T1 and T3. The micronaire value differed between T10 and many other treatments (except T5, T7, T8, and T11), while the fiber elongation rate in T10 was significantly higher than that of T1 only.

**Table 1:** Cotton fiber quality under organic fertilizer substitution for nitrogen fertilizer.

Treatment	Fiber length (mm)	Fiber uniformity (%)	Micronaire value	Fiber elongation (%)	Fiber strength (cN tex−1)
T1	27.86 ± 0.36c	81.93 ± 0.50bc	3.67 ± 0.31de	6.60 ± 0.10b	27.17 ± 0.67c
T2	28.74 ± 0.44abc	82.43 ± 0.49abc	4.04 ± 0.30bcd	6.67 ± 0.06ab	28.07 ± 0.38abc
T3	28.32 ± 0.43bc	82.63 ± 0.87abc	3.58 ± 0.19e	6.70 ± 0.00ab	27.73 ± 0.55bc
T4	28.90 ± 0.45ab	82.57 ± 0.40abc	3.57 ± 0.10e	6.70 ± 0.00ab	28.27 ± 0.81abc
T5	28.55 ± 0.33abc	83.10 ± 0.95ab	4.31 ± 0.15ab	6.73 ± 0.12ab	28.83 ± 0.81ab
T6	28.59 ± 0.30abc	82.17 ± 0.61bc	4.11 ± 0.21bc	6.73 ± 0.06ab	28.40 ± 0.46ab
T7	28.01 ± 0.60bc	82.40 ± 0.26abc	4.61 ± 0.06a	6.77 ± 0.06a	28.63 ± 0.60ab
T8	28.85 ± 0.61ab	81.70 ± 0.36c	4.63 ± 0.04a	6.77 ± 0.12a	28.33 ± 0.51ab
T9	28.72 ± 0.45abc	83.10 ± 0.56ab	3.84 ± 0.40cde	6.67 ± 0.06ab	28.73 ± 0.35ab
T10	28.23 ± 0.54bc	81.93 ± 0.25bc	4.67 ± 0.19a	6.80 ± 0.00a	28.23 ± 0.25abc
T11	29.27 ± 0.41a	83.50 ± 1.15a	4.55 ± 0.12a	6.77 ± 0.06a	29.07 ± 0.93a

Values are the mean ± standard error (*n* = 3). Different letters indicate significant difference at the p = 0.05 level.

### 3.5 Effects of organic fertilizer replacing nitrogen fertilizer on cotton dry weight and nitrogen accumulation

**Figure 4:**
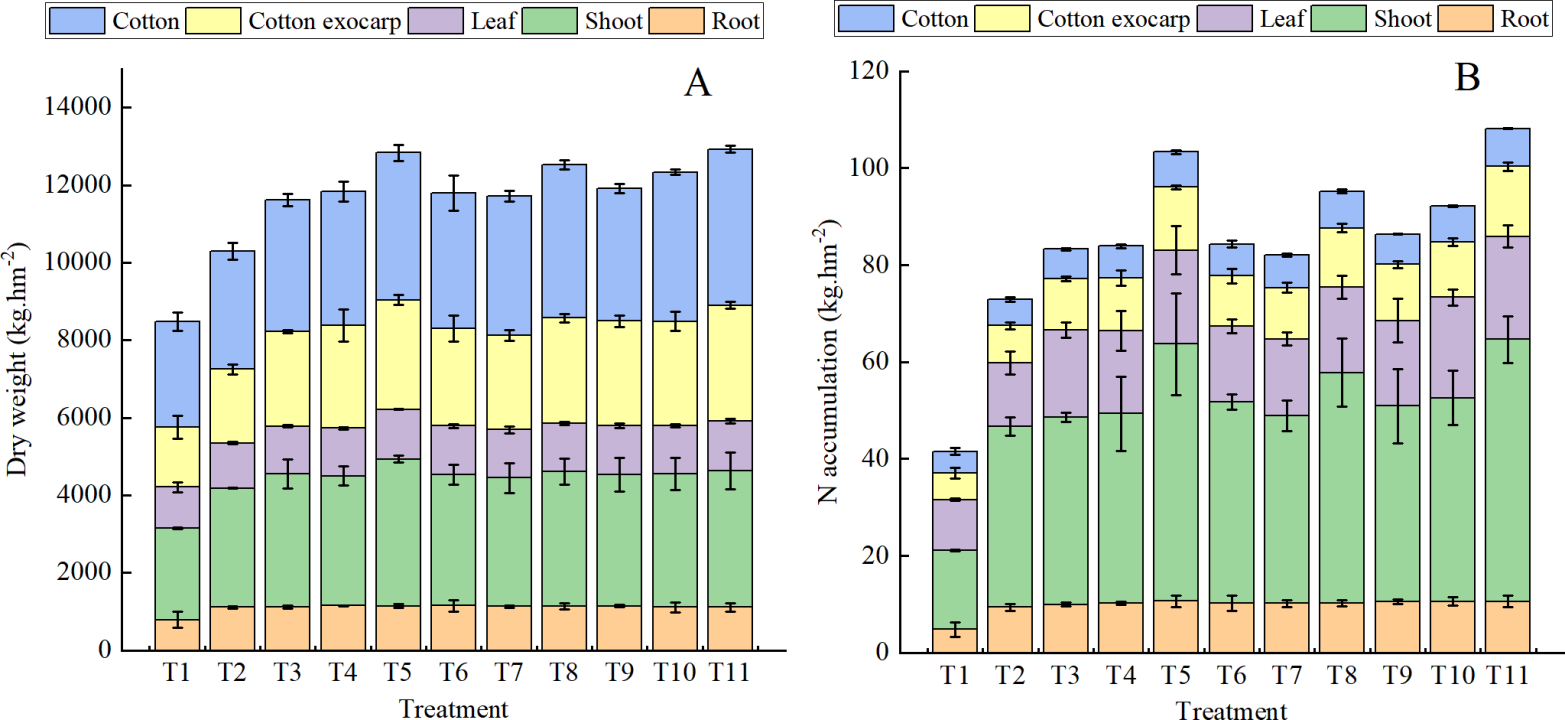
Total dry weight **(A)** and nitrogen accumulation **(B)** in drip-irrigated cotton under organic fertilizer substitution for nitrogen fertilizer.

Various combined fertilizer treatments led to significant increase in the total dry weight of cotton plants compared with T2 (**Figure 4A**). All fertilizer treatments significantly promoted nitrogen accumulation in cotton plants compared with T1 (**Figure 4B**). Applying the same type of organic fertilizers at a higher substitution ratio improved both total dry weight and nitrogen accumulation in cotton. The dry weight was mainly concentrated in the stem, cotton exocarp, and seed cotton, with a large proportion of nitrogen accumulated in the stem.

### 3.6 Patterns in plant stress with organic fertilizer replacing nitrogen fertilizer

**Figure 5:**
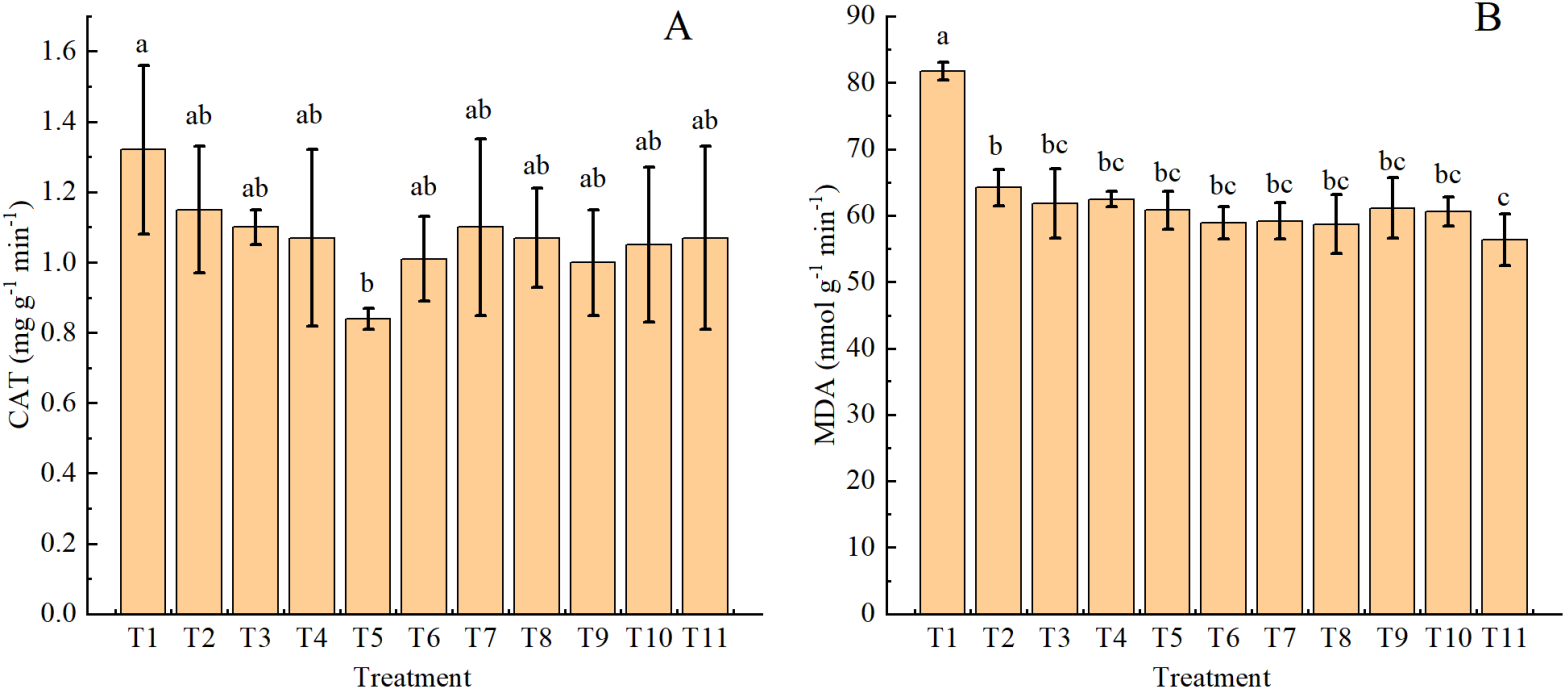
Leaf catalase activity CAT, **(A)** and malondialdehyde content MDA, **(B)** in drip-irrigated cotton under organic fertilizer substitution for nitrogen fertilizer.

Leaf CAT activity was generally reduced across all fertilizer treatments compared with T1, although the difference was significant in T5 only (**Figure 5A**). Leaf MDA content also trended lower in the fertilizer treatments compared with T1, and T11 showed the greatest effect, resulting in significantly lower MDA content than T2 (**Figure 5B**).

**Figure 6:**
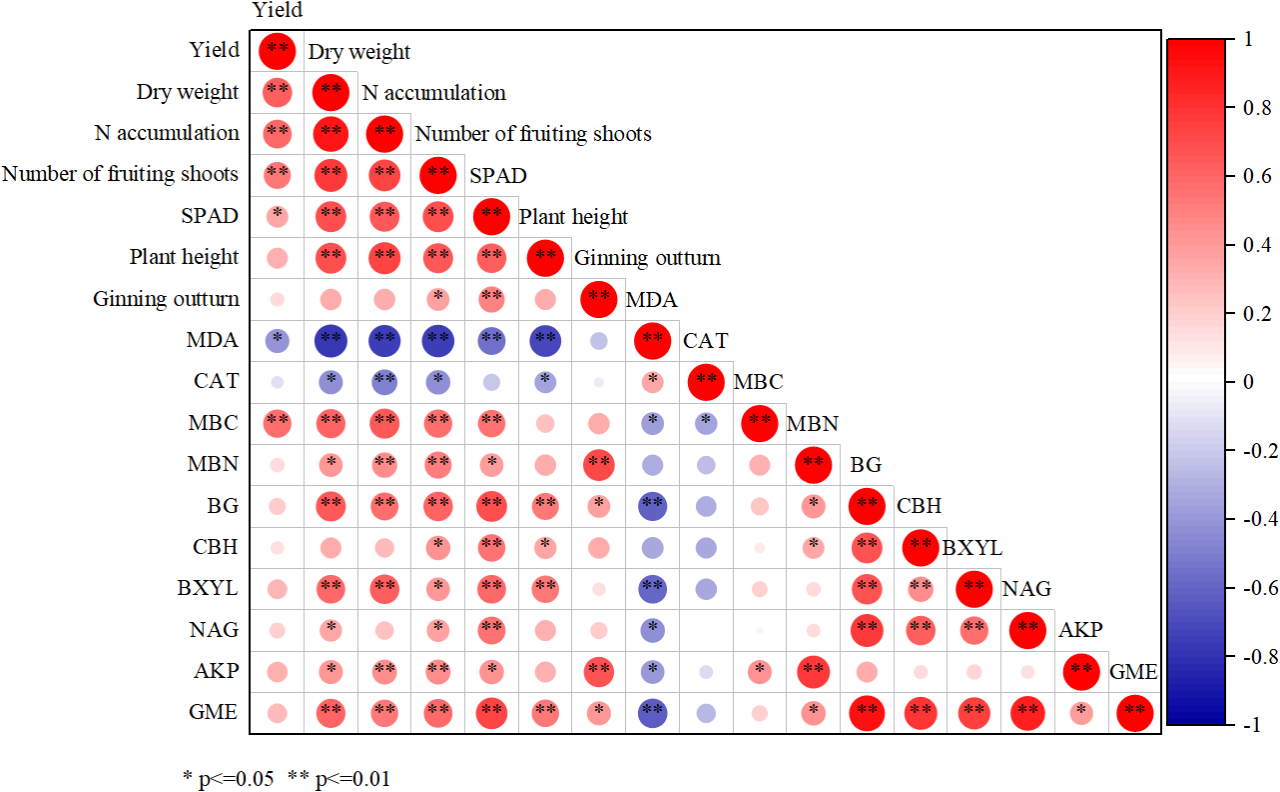
Correlation analysis of plant and soil parameters in drip-irrigated cotton. SPAD, relative chlorophyll content; MDA, malondialdehyde content; CAT, catalase activity; MBC, microbial biomass carbon; MBN, microbial biomass nitrogen; BG, β-1,4-glucosidase; CBG, cellulose β-glucosidase; BXYL, β-xylotetraerase; NAG, β-1,4-N-acetylamino glucosaminidase; AKP, alkaline phosphatase; GME, geometric mean of soil enzyme activities.

### 3.7 Effects of organic fertilizer replacing nitrogen fertilizer on soil microbial biomass

**Figure 7:**
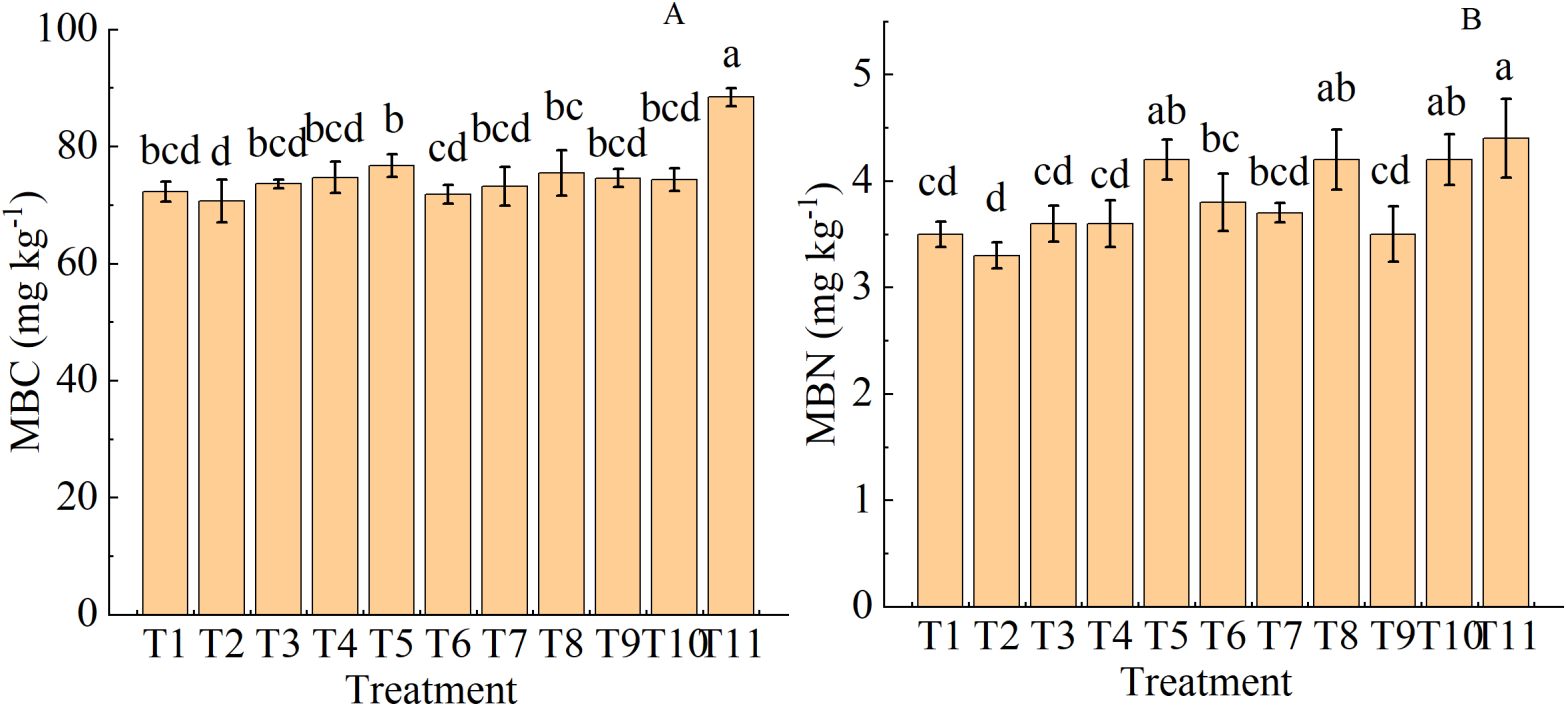
Soil microbial biomass carbon MBC, **(A)** and nitrogen MBN, **(B)** contents in drip-irrigated cotton under organic fertilizer substitution for nitrogen fertilizer.

The combined fertilizer treatments with 30% nitrogen replacement (T5, T8, and T11) significantly increased soil MBC content compared with T2 (**Figure 7A**). The MBN content was also significantly higher in these three combined fertilizer treatments than in T1 and T2 (**Figure 7B**). Both MBC and MBN contents peaked in T11.

### 3.8 Responses of soil enzyme activities to organic fertilizer replacing nitrogen fertilizer

**Figure 8:**
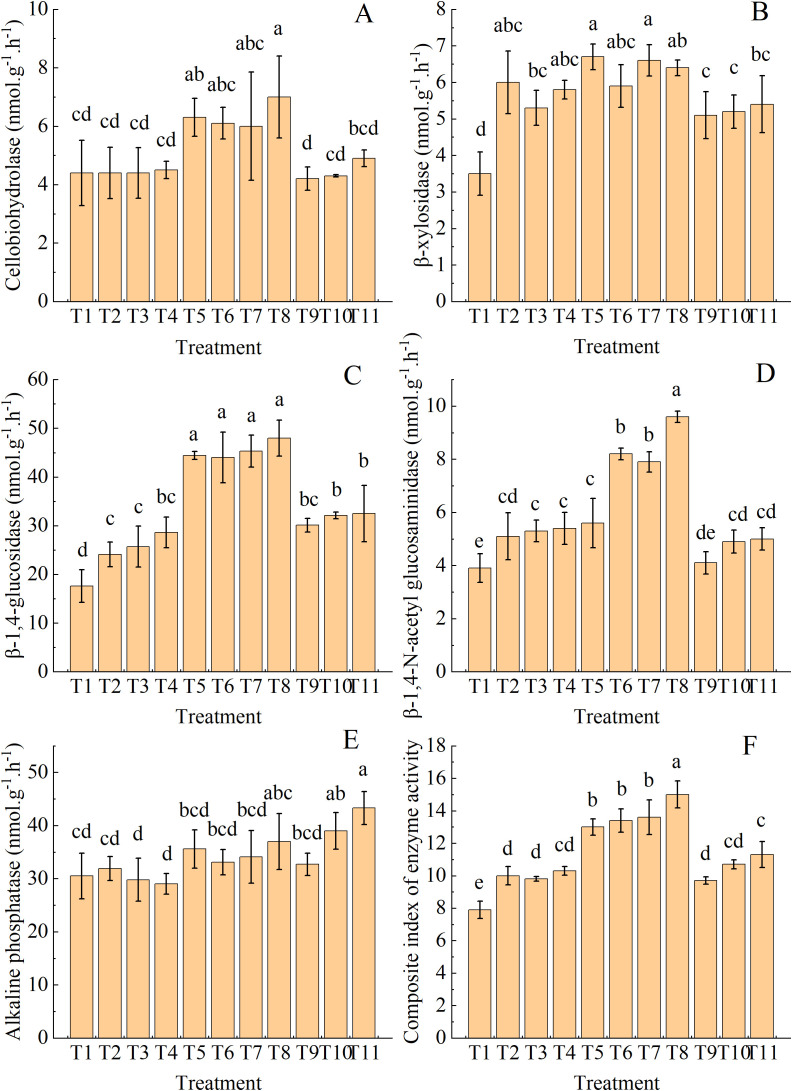
**(A–F)** Soil enzyme activities and their geometric mean (composite index) in drip-irrigated cotton under organic fertilizer substitution for nitrogen fertilizer. Note: BG, β-1,4-glucosidase; CBG, cellulose β-glucosidase; BXYL, β-xylotetraerase; NAG, β-1,4-N-acetylamino glucosaminidase; AKP, alkaline phosphatase; GME, geometric mean of soil enzyme activities.

Soil CBH activity reached its maximum value in T8, which was significantly higher than that of T1–T4, T9, T10, and T11 (**Figure 8A**). T5 achieved the greatest soil BXYL activity, exceeding that of T1, T9, T10, and T11 (**Figure 8B**). Soil BG activity peaked in T5–T8, which showed a significant difference compared with other treatments and did not vary with the substitution ratio of organic fertilizers (**Figure 8C**). In contrast, soil NAG activity was enhanced with increasing substitution ratio for the same type of organic fertilizers and a peak value occurred in T8 (**Figure 8D**). T11 resulted in the greatest soil AKP activity, which was significantly different from that of T1 and T2 (**Figure 8E**). The GME value trended upward with increasing substitution ratio for the same type of organic fertilizers and its highest level was observed in T8 (**Figure 8F**).

### 3.9 Relationships among plant and soil parameters under organic fertilizer substitution for nitrogen fertilizer

Seed cotton yield was significantly positively correlated with plant dry weight, nitrogen accumulation, the number of fruiting shoots, and soil MBC, as well as leaf SPAD value (**Figure 6**). Plant dry weight was significantly positively correlated with plant nitrogen accumulation, the number of fruiting shoots, leaf SPAD value, and plant height, in addition to soil MBC, BG, BXYL, and GME. Positive correlations also emerged between plant dry weight and soil MBN, NAG, and AKP. Plant nitrogen accumulation was significantly positively correlated with the number of fruiting shoots, leaf SPAD, and plant height, as well as soil MBC, MBN, BG, BXYL, AKP, and GME. The number of fruiting shoots was significantly positively correlated with leaf SPAD value, plant height, soil MBC, MBN, BG, AKP, and GME, together with ginning outturn, soil CBH, BXYL, and NAG. Leaf SPAD value was significantly positively correlated with plant height, ginning outturn, soil MBC, BG, CBH, BXYL, NAG, and GME, as well as MBN and AKP. Plant height was significantly positively correlated with soil BG, BXYL, and GME, as well as CBH. Seed cotton yield and leaf SPAD value were negatively correlated with MDA, while plant dry weight, nitrogen accumulation, the number of fruiting shoots, and plant height were negatively correlated with both MDA and CAT.

## 4 Discussion

### 4.1 Partial substitution of organic fertilizer for nitrogen fertilizer improves seed cotton yield and fiber quality

It has been shown that replacing 20–30% of chemical nitrogen fertilizer with organic fertilizer effectively promotes dry matter and nutrient accumulation in crops (Wei et al., 2021). This effect is related to improved nitrogen use efficiency and chlorophyll content in plants (Ren et al., 2022). Partial substitution of organic fertilizer for chemical nitrogen fertilizer has the potential to optimize nutrient utilization and promote crop yield, contributing to sustainable agricultural development (Guo et al., 2021; Liu et al., 2021b).

Organic fertilizers contain complex nutritional components, which can improve soil physical and chemical properties. For example, the application of organic fertilizers increases soil organic matter content and nutrient availability. This in turn promotes nutrient absorption and distribution by plants, thereby improving cotton yield (Shi et al., 2024). Ma et al. (2023) found that the co-application of nitrogen fertilizer and biochar enhanced nitrogen use efficiency and increased soil organic matter content, resulting in higher cotton yield. Consistently, compared with the single fertilizer treatment (T2), the combined fertilizer treatments (T3–T11) increased the dry weight of cotton plants and stimulated their nitrogen absorption, optimizing nitrogen distribution in various organs. First, the solid organic fertilizer releases nutrients slowly into the soil, exhibiting a long-lasting effect. It could continuously provide the nutrients required for crop plants during their growth period. Second, the liquid organic fertilizers contain small-molecule organic nitrogen that is readily available to crop plants. Their repeated and phased application could efficiently replenish soil organic matter, maintain microbial growth and enzyme activities, and accelerate organic matter mineralization. Moreover, drip irrigation is a key strategy to regulate the release rate and intensity of soil and fertilizer nutrients. This technique could increase the supply of soil nutrients to meet the requirements of plants across various growth stages, leading to stable, high crop yields (Elhindi et al., 2016). Therefore, the number of fruit shoots and cotton yield increased in combined fertilizer treatments, and the highest yield in T11 was 77.09% higher than that of T2. The results are in agreement with a previous study showing that the application of organic fertilizer delivered 32.28% higher cotton yield compared with the control (Chen et al., 2023).

Shi et al. (2023) showed that substituting organic fertilizer for nitrogen fertilizer improved cotton fiber quality, stem diameter and seed cotton yield. Cotton yield and fiber quality are closely related to nitrogen fertilizer management. Appropriate application of nitrogen fertilizer can increase the yield and fiber quality of cotton, while insufficient or excessive nitrogen fertilizer may cause yield and fiber quality losses (Rochester et al., 2001). However, the combined fertilizer treatments did not significantly alter fiber quality, such as fiber length, uniformity, elongation, and strength in T7, T8, T10, and T11 compared with the single fertilizer treatment. In particular, T5, T8, and T11 (30% nitrogen replacement) increased cotton yield without affecting fiber quality. Liu et al. (2023a) have established that straw application reduces nitrogen fertilizer usage and improves cotton yield, while not affecting cotton fiber quality.

### 4.2 Partial substitution of organic fertilizer for nitrogen fertilizer enhances soil microbial biomass accumulation and enzyme activities

Applying organic fertilizers can improve soil biological properties and particularly boost enzyme activities (Li et al., 2018). MBC and MBN are two major indicators for measuring soil biological fertility. The application of organic fertilizers is a potential method to rebuild microbial biomass and improve soil functions. Organic fertilizers provide abundant carbon and nitrogen sources to soil microorganisms, thereby increasing their abundances (Liu et al., 2023b). T5, T8, and T11 (30% nitrogen replacement) increased soil MBC and MBN contents by 40% and 55%, respectively, compared with T2. This result is comparable to the finding of Cerny et al. (2008) that soil MBC and MBN contents were higher after organic fertilizer application than in the control.

Soil enzyme activity plays a crucial role in nutrient transformation and cycling. Given its high sensitivity to organic matter dynamics, soil enzyme activity serves as a useful indicator for evaluating soil quality changes (Acosta-Martínez et al., 2019; Xu et al., 2020; Chen et al., 2022). There is a positive correlation between soil microbial biomass, organic matter, and enzyme activity (Nsabimana et al., 2004; Wang et al., 2022). Soil organic matter is the primary determinant of soil enzyme activity (Hendriksen et al., 2016). The composition and biomass of soil microbial communities, either directly or indirectly, are the major drivers of enzyme synthesis and production potential. During microbial decomposition, organic matter entering the soil releases nutrients, which support the growth and reproduction of microorganisms. This further enhances microbial reproduction and metabolism, thereby boosting the activity of soil enzymes (Guo et al., 2024). Among the combined fertilizer treatments, T8 was most effective to enhance soil NAG, CBH, and BG activities, while T11 resulted in the greatest AKP activity. Ren et al. (2019) also found that organic amendment increased soil microbial biomass and enzyme activity. Specifically, organic fertilizer application increased soil MBC and MBN contents, while elevating urease, protease, and arylamidase activities. Soil enzyme activity is influenced by the composition and biomass of soil microbial communities. The observed effects on absolute enzyme activity cannot be simply attributed to soil microbial biomass or the true differences in enzyme activity (Raiesi and Beheshti, 2014; Trasar-Cepeda et al., 2008). An effective method to eliminate this influence is using the comprehensive enzyme activity index, GME. The GME value of T8 was significantly higher than that of other treatments, emphasizing the overall effect of the liquid organic fertilizer 1 replacing 30% of the chemical nitrogen fertilizer.

### 4.3 Cotton growth correlates with soil microbial biomass and enzyme activities

Crop yield and plant growth are directly related to soil nutrient supply and the soil environment. The performance of plant growth parameters is also closely linked to the formation of crop yield. An appropriate nutrient supply enhances cotton yield by promoting nitrogen accumulation in plants and increasing the number of flowers and bolls, and (Qi and Zhu, 2023). Our results showed that seed cotton yield was highly positively correlated with plant dry weight, nitrogen accumulation, the number of fruiting shoots, and soil MBC, as well as leaf SPAD value. Dai et al. (2015) found that a positive correlation emerged between seed cotton yield and plant biomass under appropriate planting density. Sun et al. (2023) reported that cotton yield was positively correlated with the number of fruiting shoots. There was no direct correlation between cotton yield and soil enzyme activities in our study. However, yield-related parameters, including plant dry weight, nitrogen accumulation, the number of fruiting shoots, and soil MBC, as well as leaf SPAD, were all positively correlated with soil enzyme activities. This reflects that soil enzyme activities plays an indirect role in promoting the formation of cotton yield. In most cases, cotton yield and growth parameters were negatively correlated with leaf MDA and CAT, indicating that plant stress is a negative regulator of cotton growth and yield formation.

## 5 Conclusions

This study systematically analyzed the effects of replacing nitrogen fertilizer with organic fertilizer on cotton yield and fiber quality, as well as on soil microbial biomass and enzyme activity. The results showed that plant height, the number of fruit shoots, and seed cotton yield were all highest when the liquid organic fertilizer 2 replaced 30% of the chemical nitrogen fertilizer (T11). Applying different types of organic fertilizers at the substitution ratio of 30% showed comparable effects on seed cotton yield across T5, T8, and T11 (6243–6933.47 kg ha^-1^), which exceeded that of the single fertilizer treatment (T2, by 59–77%). T11 contributed to the greatest fiber length, uniformity, and strength, while T5, T8, and T11 increased soil microbial biomass carbon and nitrogen contents. The combined fertilizer treatments also enhanced soil enzyme activities of β-xylotetraerase (T5), β-1,4-N-acetylamino glucosaminidase, cellulose β-glucosidase, and β-1,4-glucosidase (T8), and alkaline phosphatase (T11). T8 exhibited a higher positive effect on the comprehensive enzyme activity index than the other treatments.

In conclusion, T8 and T11 with the liquid organic fertilizer replacing 30% of the chemical nitrogen fertilizer are the optimal fertilization treatments for cotton planting in Xinjiang. These two treatments can improve cotton yield and soil enzyme activity while ensuring fiber quality. However, the results are obtained from a one-year field trial, and multi-year trials are needed to verify this conclusion.

## Data Availability

The raw data supporting the conclusions of this article will be made available by the authors, without undue reservation.
